# Effect of Formulation Variables on Rifampicin Loaded Alginate Beads

**Published:** 2012

**Authors:** Kishore Narra, Unnikrishnan Dhanalekshmi, Govindaraj Rangaraj, Devendiran Raja, Celladurai Senthil Kumar, Pully Neelakanta Reddy, Asit Baran Mandal

**Affiliations:** *Bio-Organic Chemistry Laboratory, Central Leather Research Institute, (Council of Scientific and Industrial Research) Adyar, Chennai-600020, India. *

**Keywords:** Rifampicin, Sodium alginate, PVP, PVA

## Abstract

The present work investigated the preparation of biodegradable beads with alginate polymer by ionotropic gelation method to improve the control release properties of the antibiotic rifampicin. Ionotropic gelation method was applied to prepare beads using calcium chloride (CaCl_2_) as cationic component and alginate as an anionic component. In this method, adding 0.5% w/v polyvinyl alcohol (PVA) to sodium alginate (3.0% w/v) and 2% w/v of polyvinyl pyrrolidone (PVP) to the CaCl_2_ solution were maintained to study the drug-loading and its released characteristics. The results showed that the addition of PVA and PVP significantly improved drug-loading, encapsulation efficiency and release characteristics. This demonstrates that the ionic gelation of alginate molecules offers a flexible and easily controllable process.

## Introduction

Tuberculosis (TB) is a chronic communicable disease caused by the bacterium Mycobacterium tuberculosis that infects over 1.8 billion people worldwide and is responsible for 1.5 million deaths annually (1). Treatment of tuberculosis is generally successful, except in the case of multiple drug-resistant strains of mycobacterium tuberculosis. Rifampicin (RIF) is a first-line drug for being used in the therapy of tuberculosis and is included in the list of recommended drug regimens for the treatment of latent M. tuberculosis infection in adults **(**2). 

Rifampicin is the semi-synthetic hydrazine derivative of Rifampicin B **(**3). In the last several years, many different types of rifampicin controlled release formulations have been developed to improve clinical efficacy of drug and patient compliance. 

Beads are one of the multiparticulate delivery systems and are prepared to obtain prolonged or controlled drug delivery, to improve bioavailability, stability and target drug to specific sites. Beads can also offer advantages like limiting fluctuation within therapeutic range, reducing side effects, decreasing the dosing frequency and improving patient compliance (4)**. **

In view of biocompatibility, a natural polymer like sodium alginate has been used in drug delivery applications. Sodium alginate (SA) is a sodium salt of alginic acid, a naturally occurring non-toxic polysaccharide found in brown algae. It contains two uronic acids, viz., *α *-L-guluronic and *β*-D-mannuronic and is composed of homopolymeric blocks and blocks with an alternating sequence (5). Gelation occurs by cross-linking of the uronic acids with divalent cations, such as Ca^2+^. This phenomenon has been used to prepare an alginate bead for drug delivery system. The formation of calcium-alginate beads by ionotropic gelation was achieved by dropping the drug containing sodium alginate dispersion into a CaCl_2_ bath (6, 7). 

Drug released from calcium-alginate beads depends on the diffusion of the drug in the gel matrix (7). The release characteristics of the entrapped substances could be improved by surface complexation of alginate with water-soluble polymers into the beads, such as chondroitin sulfate (8).

Polyvinyl Alcohol (PVA) has been used in drug delivery applications because of its desirable properties such as nontoxicity and non-carcinogenicity. PVA is a highly hydrophilic polymer and has poor stability in water. To overcome this problem, PVA should be insolubilized by blending, copolymerization, grafting, and cross-linking. The polymer blending technique can be considered as a useful tool for the preparation of new alginate beads with PVA **(**4).

The choice of Polyvinyl Pyrrolidone (PVP) was due to its long-standing and safe record in biomedical/pharmaceutical application. Additionally, it has been reported to enhance drug circulatory time in plasma when used as delivery system (9).

In this study, we prepared sodium alginate beads using rifampicin as a model drug. The main objective of this study was to prevent drug leaching during preparation and to improve the drug release rate. We studied the addition of 0.5% w/v PVA to the sodium alginate solution, and the addition of 2% w/v PVP to the CaCl_2_ solution on the drug-loading and also *in-vitro *release characteristics.

## Experimental

*Materials*

Sodium alginate was purchased from Himedia laboratories (Mumbai, India). Rifampicin, polyvinyl alcohol (PVA) and polyvinyl pyrrolidine (PVP) were obtained from Sigma Aldrich chemie GmbH, (Sigma Aldrich, Germany). Calcium chloride (CaCl_2_) was purchased from Rankem laboratories (Mumbai, India).

*Preparation of Rifampicin-alginate beads*

Three % w/v of Sodium alginate solution was prepared in 25 mL of deionized water under gentle mixing while heating. Two hundred and fifty mg of rifampicin was added to this sodium alginate solution and stirred using an over head stirrer (Remi instruments, Mumbai) for 5-10 min at 1000 rpm to obtain a homogenous mixture. The mixture was kept aside until the air bubbles disappeared completely and then it was extruded dropwise into 50 mL of 1% calcium chloride solution through the 26 gauge needle (F-1 beads). Similarly the F-2 beads were prepared by incorporating 0.5% w/v PVA into drug-alginate mixture and extruding dropwise into 50 mL of 1% CaCl_2_ solution. F-3 beads were prepared by extruding the drug-alginate mixture dropwise into the 50 mL of 1% CaCl_2_ solution containing 2% w/v of PVP. F-4 beads were prepared by incorporating 0.5% w/v of PVA and drug-alginate mixture into 50 mL of 1% CaCl_2_ solution containing 2% of PVP. The differently prepared formulations were presented in Table 1. The gel beads were cured in gelation solution for 1 h, then filtered, and rinsed several times with distilled water and dried at 45°C for 12-16 h in hot air oven.

**Table 1:** Formulation design of Rifampicin loaded sodium alginate beads

**FORMULATION CODE**	**SODIUM ALGINATE (%)**	**DRUG (mg)**	**CaCl**_2 _ **(%)**	**PVP (%)**	**PVA (%)**
F-1	3.0	250	1	---	---
F-2	3.0	250	1	---	0.5
F-3	3.0	250	1	2	---
F-4	3.0	250	1	2	0.5

*Fourier transforms infrared measurements (FTIR)*

The drug and polymer interactions were studied by infrared spectroscopy. The IR spectra were recorded in the wavelength region 400-4000 cm^-1^ for pure rifampicin, sodium alginate, plain alginate beads and Rifampicin loaded alginate beads using Thermo Nicolet, Avatar 320 (USA) instrument. 

*Scanning electron microscope (SEM)*

The morphology and surface structure of the beads were observed using SEM photographs taken with Jeol JSM-6360 instrument. The beads were made conductive by sputtering thin coat of platinum under vaccum using Jeol JFC-1600 autofine coater and then the images were recorded at magnification of 80x.

*Determination of drug content of the beads*

The known amount of beads was crushed in a mortar with a pestle and transferred into a beaker containing 100 mL phosphate buffer with pH of 7.4 and stirred using overhead stirrer for the complete swelling and bursting of the beads, then the solution was filtered through 0.45 μm membrane filter and the concentration of drug in the solution was determined after appropriate dilution through phosphate buffer with pH of 7.4, using UV spectrophotometer (Perkin Elmer) at 475 nm. The drug-loading and percentage of entrapment efficiency was then calculated as the formulas indicate.

$\mathrm{percentage entrapment efficiency}=\frac{\mathrm{Practical drug loading}}{\mathrm{Theoretical drug loading}}\times 100$

$\mathrm{percentage drug loading}=\frac{\mathrm{Practical drug loading}}{\mathrm{Theoretical drug loading}}\times \mathrm{100}$

*In-vitro drug release*

The *in-vitro *drug release from the beads was studied in buffer with pH of 1.2 and 7.4, using shaking incubator. The known quantity of alginate beads (100 mg) from each formulation was transferred into conical flasks containing 100 mL of buffer solution and the flasks were shaken with a rate of 50 rpm at 37°C. The samples of 5 mL aliquots was withdrawn at predetermined time intervals and the same volume of fresh preheated buffer medium was replaced into the conical flasks to maintain sink condition throughout the experiment. The withdrawn aliquots was filtered and analyzed for drug content using UV spectrophotometer at 475 nm after suitable dilution with respective buffer solutions.

## Results and Discussion

*FTIR studies*

**Figure 1:**
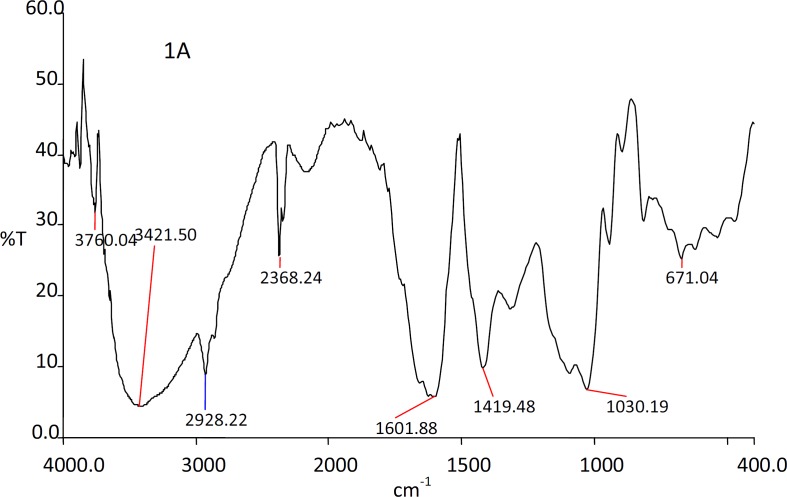
IR spectra of sodium alginate

**Figure 2:**
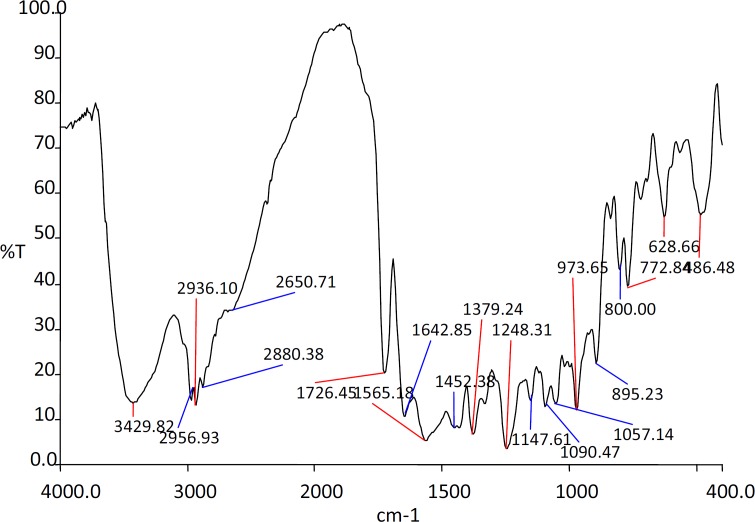
IR spectra of Rifampicin

**Figure 3:**
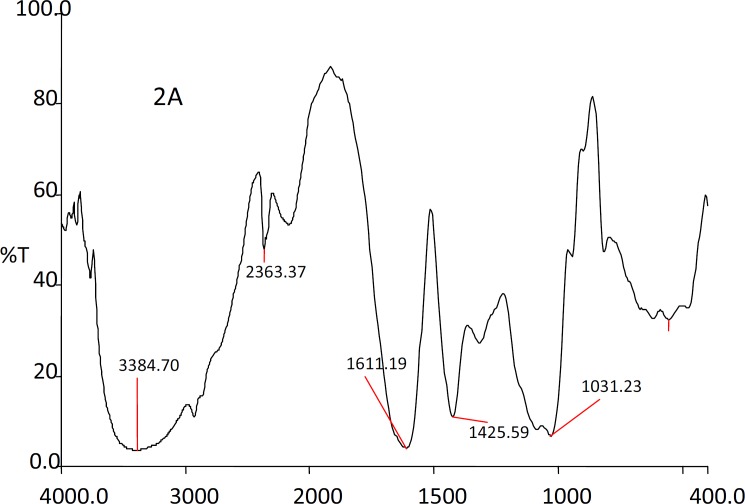
IR spectra of sodium alginate beads

**Figure 4:**
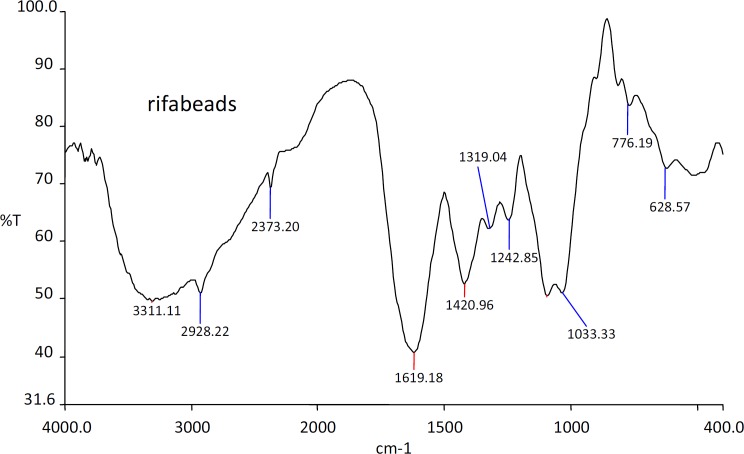
IR spectra of Rifampicin loaded sodium alginate beads

The IR spectra of the substances used in the formulation and the prepared beads were shown in the Figures 1, 2, 3 and 4.

The IR spectra of the Rifampicin formulated beads’ peaks were at, 2928.22, 2373.2, 1420.9 and 1033.33 cm^-1^ corresponding to alginate and also at 3311.11, 1619.18 and 1242.85 cm^-1^ corresponding to the drug. This confirms that there was no interaction between the drug and the substances used in the formulation.

*SEM analysis*

**Figure 5:**
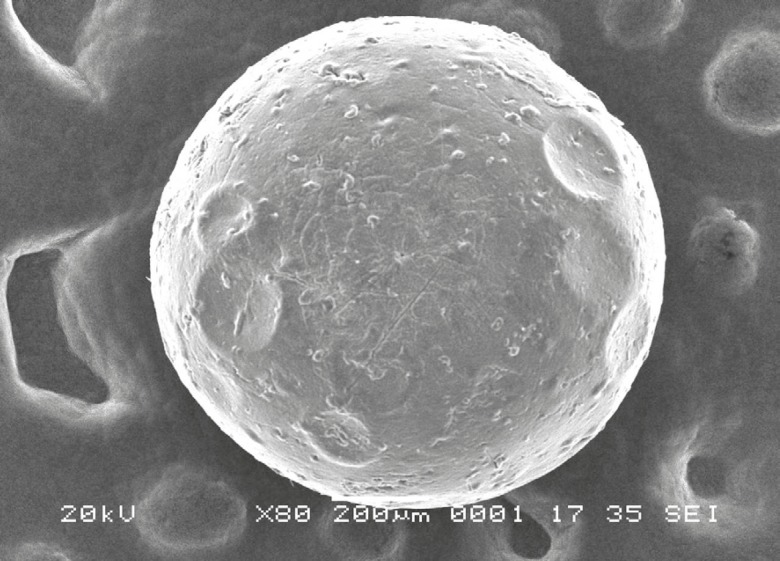
SEM photograph of Rifampicin loaded bead (F-4) at 80x

A SEM photograph of formulation (F-4), a single bead taken at 80x magnification, was shown in Figure 5. As seen from the figure, the drug loaded beads were almost of spherical in shape and have a rough surface.

*Drug-loading and entrapment efficiency*

The drug-loading and entrapment efficiency of different formulations were shown in Table 1. The formulation (F-4) with 0.5% w/v PVA and 2% w/v PVP shows better drug-loading efficiency compared to the other formulations.

*Effect of PVA on drug-loading and entrapment efficiency*

The addition of PVA to the drug-alginate mixture solution (F-2) resulted in a large increase of loading and entrapment of drug in alginate beads. This may be due to an increase in viscosity of the bead preparation solution. As a result, alginate trapped more drug molecules and so loading and entrapment efficiency increased compared to the absence of PVA in drug-alginate mixture (4).

*Effect of PVP on drug-loading and entrapment efficiency*

The addition of PVP to the cross linking solution (F-3) has shown significant increase in the drug-loading and entrapment efficiency. This may be due to the increase in viscosity of the cross linking solution by PVP, so that it may block the pores of the alginate beads and hence it may prevent the drug leaching to the cross linking solution.

*In-vitro drug release*

**Figure 6:**
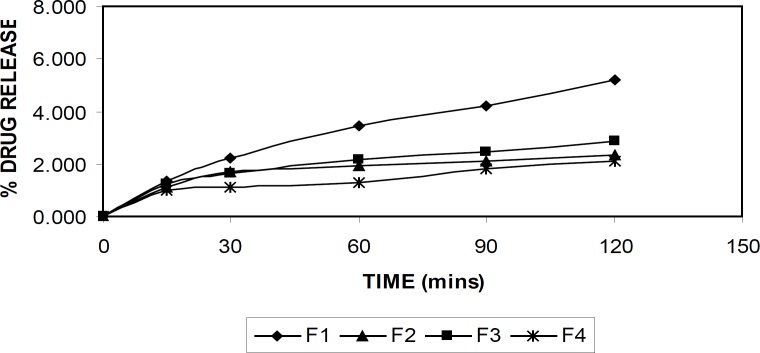
In-vitro Drug release at pH of 1.2

**Figure 7:**
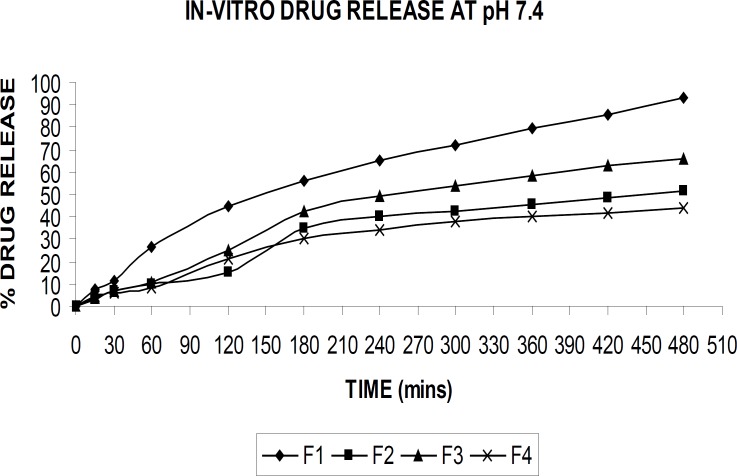
In-vitro Drug release at pH of 7.4

The drug release from the alginate beads were studied in buffer solutions with pH of 1.2 and 7.4 at 37°C. Figures 6 and 7 demonstrate the drug release in respective buffer solutions from different formulation. A pronounced difference was observed in the release data between pH of 1.2, and 7.4. The formulation (F-4) showed the better sustained drug release was achieved compared to the other formulations. 

At acidic pH, rifampicin release from gel beads was low (1-5%) in all formulations for 2 h. At low pH values, the less swelling should reduce the matrix permeability and limit the drug diffusion (4)**. **At this pH, alginate was protonated into insoluble form of alginic acid which displays properties of swelling and explained the low amount of release. An increase in the release rate (40-90%) was observed at pH of 7.4 in 8 hr. At pH of 7.4, the deprotonation of alginic acid caused disintegration of the bead systems and nearly completed the release of rifampicin as soluble molecular form. 

When PVA was added to the drug-alginate mixture (F-2), rifampicin release rate was decreased as it is seen from the Figures 6 and 7. Alginate is a natural water-soluble polymer and contains hydroxyl and carboxyl groups, which imparts hydrophilicity to the molecule. On the other hand, PVA is virtually a linear polymer with a small hydrated volume compared to alginate and thus PVA produces a compact network of macromolecular chains in the blend beads. Therefore, penetration of liquid molecules through PVA contained drug alginate beads and then diffusion of drug to external medium is difficult compared to the drug-alginate beads (4). 

The carbonyl group of PVP has a high molecular weight of approximately 1,000,000 Da and it is hypothesized that diffusion of the PVP within the bead is inhibited by its high molecular weight. The presence of PVP in the formulation leads to a modulation of the drug release from the ‘pseudo-gel layer’surrounding the bead, which controls the drug release rate (10).

## Conclusion

The rifampicin loaded sodium alginate beads were prepared by ionic gelation method. In this method, various formulation variables such as the addition of PVA to the drug-alginate mixture and addition of PVP to cross linking solution were studied. High drug-loading, encapsulation efficiency and sustained drug release were obtained in F4 formulation. Previously, no substantial work have been reported by employing PVP in the cross linking solution. Hence, PVA and PVP can be used in the formulation to improve *in-vitro *characteristics of rifampicin loaded alginate beads 
